# Enforced Climate and Diet as Affecting the Teeth

**Published:** 1889-01

**Authors:** J. T. Codman

**Affiliations:** Boston, Mass.


### ENFORCED CLIMATE AND DIET AS AFFECTING THE TEETH.*

BY J. T. CODMAN, D.M.D., BOSTON, MASS.

All problems relating to a solution of the causes of the decayed teeth of the human race are worthy of most thoughtful consideration.

More particularly should they interest us, as the object of our calling is especially for their care, preservation and restoration.

In offering to you this humble tribute of thought, it is my hope that it may awaken an impulse to search with greater scrutiny for causes hardly yet investigated and certainly not yet brought under universal consideration.

If I begin by saying that harmonious physical surroundings and harmonious diet with proper heredity should produce perfect men, women and children, you will probably agree with me; and will say that this result will follow as naturally as cause and effect; that is, given healthy, prenatal and antenatal conditions there would be universal health and no such thing as decay of the human teeth.

From this logical conclusion I think there is no appeal unless we believe ourselves and the creative power to be illogical, and that our reasoning powers are given us to confuse, beguile and perplex us instead of to comfort, strengthen and sustain us.

Following this line of thought let us see where we shall be led by it.

Let me repeat the conclusion, that harmonious conditions will bring forth the well-developed undiseased man; but as we look about on every side what do we see? Instead of a race of idealy perfect beings, only an undeveloped and largely a physically and mentally diseased race, and as we know that man has made but few of his mental and physical surroundings, we conclude that his surroundings have mainly made him; and that they must be to a large extent inharmonious, for had they all been harmonious perfect harmonious beings and healthy races would have been created from their influences.

The general thought that runs through Christendom and has given tone to our ideas and a bias to our minds has been taken from the Jewish and Assyrian theologies, how much farther back in time no one knows. It is of a perfect creation—a tempter, a fall, and a degenerate race because of that temptation and fall.

---

* Read before the New Jersey State Dental Society at its Eighteenth Annual Session held at Asbury Park, July, 19, 1888.

Were this doctrine as accepted entirely true, there would be no need of farther search for primary causes; but true science, I maintain, starts from the beginning of things in its investigations; therefore, no old document, wherever it may be found, should be accepted as true without close scrutiny. Its theories and probabilities should be discussed; its facts brought out and weighed in the full electric light of modern science before judgment should be rendered on it. The wise men of to-day differ from the wise men of centuries past, in that they have found thousands of new facts to guide them to their conclusions.

It has been considered scientific to say that man is an omniverous animal; but, glancing at the structure of his body, it becomes a question of whether the condition in which you find him as an omniverous eater is a true and normal one.

Certain well-known facts regarding his physical formation, such as the long intestine, the delicate and non-pointed teeth, the weak-muscled jaw and its lateral motion, which belong solely to the graminiverous animals. have led many profound students of physiology to believe that normally man is not an omniverous eater, but one in whom the frugiterous and graminiverous instincts prevail.

If the race of man is not *per se* omniverous, and eats omniverously, such a condition must bring about deplorable results from the violation of one of the primary conditions of health; that is, strict obedience of the physical laws of diet.

Let us now glance at the condition of the earth and its relation to food supply. A new-born child is not responsible for the country into which it is born, whether it is Lapland, Siberia, Brazil or Honduras—whether Italy, France, the United States or elsewhere; but it is evident that if born among the Esquimaux its diet after it leaves the breast cannot be like that of the child born in sunny France or in Brazil. There will be found no whale-blubber in the French Alps or pineapples in Lapland; yet the child born in either country will live and grow to maturity and old age.

Man is the choicest product and the most helpless of God's creation at birth; but finds protection provided for him in the parental instincts implanted in his progenitors. Launched amid wild and savage beasts, weak, feeble and naked, he finally feeds, clothes and defends himself in spite of his delicate organism by the cunning of his brain and hands, and subdues the forces of nature to his uses. Before he learned to build houses, if there was such a time, he hid himself in caves to protect himself from storms of wind and rain, or he wove the branches of trees together for a habitation, or he climbed into a high tree top, where no wild beast could follow, for security and a home.

As we go back in the ages, assisted only by the light of the imperfect science of to-day, we find phases of man's existence that are clouded in doubt and darkness; and generations will probably have to study problems of geology, cosmogony and anthropology before the question of what is our normal race condition will be positively settled; but, personally, I am satisfied that the earth's condition at a time not beyond tradition was very different from the present in its relation to food supply. That the earth at one time was covered with verdure from pole to pole; that the elephant and the mammoth dwelled once in the umbrageous shades of the polar region and meandered amid its giant trees, hardly admits of a doubt. From the nature of its vegetation, the remains of which are still found, the climatic temperature must have been mild, and a more equable climate extended over the whole earth than at present exists.

Since that period, at some unrecorded time, an era of intense cold has passed over the earth. Whole continents have been buried in unmeasured depths of snow and ice. Geologists call it the "ice age." From the north pole to the equator, adown the valleys of Labrador and adown the valleys of Brazil alike, have moved the ice glaciers. The fearful cold winds have utterly destroyed all the vegetable and vegetating food for man, except, possibly, in some of the now called torrid regions. Doubtless the race was depleted; doubtless cold, hunger, poverty and death reigned everywhere, and the wild beasts, made doubly savage by famine, preyed on the living and the dead.

By what tremendous catastrophe or cataclysm this event came about we do not know. How many ages poor humanity wandered over the earth in a half-starved condition no one knows.

Ignatius Donnelly, in his interesting book, entitled "Ragnarok," theorizes that some cometic body or disrupted planet, coursing through the immense spaces of the heavens, came so near as to rain showers of boulders, stones and sand on us in immense quantities, and heated the waters of our oceans and rivers so hot by its contact that immense quantities of vapor were carried into the upper heights of the sky, and falling again, as fall it must, in snow and cold rains, the temperature of our little earth, only 25,000 miles in circumference, was so thoroughly cooled that it has not yet returned to its normal heat by the slow warming process of the sun's rays.

It is the fashion of our day to cry down Donnelly on account of his attempt to prove that Bacon was the author of Shakespeare's plays, but we must not forget that it takes an ingenious and a searching mind to make a plausible plea to that effect; and no man can read "Ragnarok" without admiration of the thought and research spent by the author of that remarkable work, and I commend it to you as highly interesting, and a book to awaken thought and investigation into world problems.

The immense littleness of our globe, in comparison to the greatness of some of the planets and fixed stars, should teach us all to be humble. Vagrant wandering comets have been chronicled whose sizes have been so large that our earth would make but a scarcely perceptible pinhole in passing through the immense train that swept by, measuring a hundred million miles in length.

Taking, then, our human anatomy; taking man as a fruit-eater; as a nut and a grain-eater; as a lover of the beautiful things that smell sweet and are luscious—a lover of strawberries and grapes; of those fruits that delight the eye—the peach, the pomegranate and the orange; that are sweet to the taste, as the date, the fig and the banana, as well us many tropical fruits unknown to us here. Take him as a lover of beautiful flowers that nourish the blood as well as the mental organism. Take him even as a lover of eggs and the birds that lay them, as a partial flesh-eater, if you please, and decide, as you have a right to, that this combination of bread and meats and fruits with a genial climate will give healthy blood, bones and muscles.

Put this organism, this spiritual being—by that I mean a being with high instincts and tastes, soaring upward, restless, desiring cultivation, and not satisfied with a merely animal life—on an ice plateau, with ten months of winter and four to fourteen feet of snow and ice, with a diet of raw fish and clams and nothing else, and what would become of him? What sort of a grand man would he make? Why like the present Esquimaux, a man without influence, power or nationality.

Let us look at the reverse. As we approach the bread-eaters, notably the Teutonic and Slavonic races, how rapidly power increases, how rapidly intelligence increases, how ingenuity thrives, how muscular the men grow, how active their brains grow, how the nation and the arts of civilization thrive.

Starting, then, at a point of time more or less remote, with a salubrious climate and a bountiful, fruitful vegetation, what would be the gravitation of the race towards the destruction of beasts and the feasting on their carcasses, or towards the cultivation of the beautiful fruitage that begins in leaves and flowers and ends in tempting clusters of grape and grain? Towards the latter, I think.

And the reason why I think so is from my observation of the present tendencies of our people. Picture to yourself the average diet of New England people two or three generations ago. It was of bread made of Indian corn, rye and occasionally barley and wheat, dry beans and peas, cabbages, squashes, pumpkins, parsnips, turnips, carrots and onions, apples and hard pears. For meats, beef, pork, poultry, mutton, wild game and fish. T ese were the principal foods of a family for nine months of a year. For sweetening they had molasses and a very little sugar; and for drink, apple cider, rye coffee, rum and water, milk, some genuine coffee and very little tea.

When "killing time" came, the family hog was slaughtered. A large portion of it was put in salt brine; some parts of it were smoked and some made into sausages. It was the same with the cow or ox that was killed. A part was sold to friends and neighbors and the remainder corned, and a good stock of salt codfish was laid in for winter's use.

All this is well known, but such great changes have taken place since then that it is worth while to dwell a moment on them Wheat, finely ground, has largely taken the place of Indian corn. Oat meal is largely used where once it was not. Rice, sago, tapioca, and various preparations, as farina and grannum, are consumed. Potatoes are enormously used, where seventy-five years ago they were hardly known. Tomatoes, now in general use, were then unknown. Numberless varieties of all the vegetables of those days have been added to our list, such as the horticultural seavey and Lima beans, toothsome peas, new varieties of sweet corn, turnips, onions, squashes, cauliflowers, celery, etc., etc. Varieties of apples have reached the hundreds, and pears also. The tiny strawberry has grown to be a mouthful each. The Lawton blackberry, found in Dorchester, a part of Boston, is a wonder in size and flavor, and is not beyond the ability of the average family in cost. The cultivation and production of fruit and vegetables in our orchards and gardens is enormous, and as our seasons are not long enough to supply the demand, we start our crops largely under glass; and we make our fruit season longer to the consumer by "cold houses," where we keep it into the winter. Ready transportation brings us strawberries from Florida and from Nova Scotia in the same spring and summer months.

From the South early spring brings us all kinds of young vegetables, and summer follows with early fruits, and then melons and peaches, and still the demand increases year by year. From foreign lands come grapes, figs, oranges, bananas, dates, prunes, olives, lemons, and nuts, increasing in quality and quantity every season.

These give our people a different and, I believe, a better diet than formerly. Add also to this the immense quantity of canned fruit and vegetables that are used, and we can see readily that the dietetic change that has taken place, and is taking place in our people, is very great and remarkable.

The diet of former years had its result. Transportation was difficult and slow, and as the winter progressed, dinners as well as as other meals were monotonous, being largely of corn-bread, salt pork, salt beef, salt fish, and winter vegetables. Cabbage, beets, carrots, and parsnips were boiled with the meats. Pork was fried and hashed with salt fish; but down they all went. Hard work made it possible to digest them, greasy as they were; and they washed them down with a daily ration of diluted rum and molasses, and plenty of cider. Such is a rough, but I believe a true sketch, of a former New England diet.

One of the great departures from earlier diet lies in the less use of fat. Economy was a habit and a teaching of the people, and nothing was to be wasted, and so in order to eat the whole animal they made suet puddings, and often flavored them with wild tanzy weed. They gloried in fried pork, and fried beef, and fried doughnuts. Every thing was rich with gravy. Meat was minced with condiments, with a good proportion of suet, and made into pies, and the very word *rich* indicated to the minds of the people something greasy and fat, and they *thoroughly agreed* with:

"King Arthur who a pudding did make,
And stuffed it well with plums;
And into it put great lumps of fat
As big as my two thumbs."

Contrast the ideal of those days with the charming cookery of our best hotels and houses of to-day. There everything is free from that kind of grossness. What our people have taken from one side they have placed on the other. They have banished the fat and put the fine growths of the summer and autumn in its place, and this is, I believe, a natural tendency towards the desirable and normal diet; and with this banishment has vanished the severity of a large class of diseases, and notably with gross feeders, the gout.

But we may ask how is it that man has the almost universal flesh eating tendency if he is not normally a flesh eater? We find him a promiscuous feeder and yet my idea is that he is not naturally omniverous; but if not, where did he get his omniverous desires.

The reply is that his surroundings have made him what he is, or rather his past surroundings have given him the tendencies and habits that still cling to him and yet remain, it may be, for centuries longer.

Believing as I do that the conditions under which the human race found itself during the age of ice, the succulent vegetation was blighted and humanity was fast drifting back towards savagism from which it may have originally sprung, there must have been a time when little other food but animal could have possibly been obtained and the human race *had* to eat what it could get to eat.

The severely cold age must have existed many centuries and man must have gone as far from the normal condition of diet as the climate varied from its normal condition of food production.

Had humanity, had human beings been beings of the lower order like cattle or sheep they would have perished "like cattle," as we say, from the fact that the lower races have not the protection placed around them that man has, and are not able to withstand marked changes of diet. But man with his wonderfully adaptive digestive apparatus by becoming omniverous, or, in other words, by feeding upon any and everything he could find to eat, fish, flesh or fowl, anything flying in the air; anything living in or on the earth; anything in the waters or on the shores along side of them, kept alive the human race to fulfil the destiny appointed for it. And it survived, when many of the lower races perished forever.

Mankind is slow to move out of the ruts it gets into, be they what they may, of kingcraft, of priestcraft, of witchcraft even. It rocks its babies in the same kind of cradles it did hundreds of years ago; its household utensils are the same; its traditions are the same, and likewise its diet is the same. Only among the pushing bread eaters, the restless and ambitious white races forfeited by a destructive religion has progress been made, but that progress comes slowly. Man, as has often been said, is a creature of habit, and there is a constant contest between the forces within with their normal tendencies and the habits forced on him by his outward surroundings.

We all know what is the normal diet of an infant as it lies on its mother's breast, in the first few months of its life; but what will circumstances force on it as soon, or even sooner than it leaves its mother's milk? It may be its mother gives it a meat-bone to suck; or may be it fills its mouth with blubber oil; or it may be she gives it oatmeal or rice, or potato and garlic; and for its drink she gives it, may be, cows' milk flavored with coffee, or gives it a sip of beer "to make it a German," or a little tea because "its mother likes it," or the sugar from the bottom of the wine-glass, because the "father likes it."

And as the child grows older, its mother prepares its food for it; and she greases its potato, and she salts its potato, and she peppers its potato. She mustards his meat, and fries him a doughnut, and gives him some mince pie. She sets his morning cup of coffee before him only a trifle weaker in strength of decoction than his father's, and gives him hot batter cakes and fine flour bread, soda raised; and does all she can to make a man of him.

And where will he land? As a man of diet on his father's and mother's platform. Only he will have poorer health, poorer teeth and a poorer stomach; and at the end of a few years will have assisted the good doctor towards paying for his carriage, or the good dentist for filling his honeycombed teeth, or the good sexton for his children's funerals. Tell me, if you can where, in all this do the inherent instincts of his nature have a fair chance to show themselves? And tell me also if there is not here some chance for a science of diet to interfere!

We need science at the base of diet, and its proofs must rest in physiology, and not on the habits of men that may be forced on them by their various surroundings. In anticipation of this science, I suggest this axiom as one to study—one to prove or disprove:

*The normal diet of man will keep his teeth clean without assistance from powder, soap or brushes.*

If this is so, we have something to guide us towards the great end we strive for: a knowledge of a basic fact. The animal, led by its instincts and given little power to vary from a strict course of diet, must obey, and is found everywhere in its native haunts with excellent teeth, and largely so in its domesticated state. Its breath is sweet, and it pays no doctor's bills. Why should not man's teeth be good, living on a proper diet?

I will formulate another axiom: *All food eaten should be soluble in the saliva.*

That is, it should be so soluble that, after eating, the tongue and the movable muscles of the mouth, with the saliva, shall be able to wash and brush the teeth free from all sticky particles of food by their automatic motions.

This seems, on the face of it, to be true; because it is so with all the lower animals without exception. Even your horse, your dog, or your cow, stalled as they may be, hardly present an exception to it.

I do not now propose to follow out the results of the application of these maxims to daily life; but they will debar from use considerable of the food now consumed, and, as I believe, the portion that is unnecessary and unwholesome.

As surely as you know the dietetic habits of an individual or a race, so surely should you be able to diagnose their condition of progress. Take our savage tribes and analyze them. Raw or half-cooked meat, pounded maize, a wigwam, a few pine-knots for light, and a pipe of tobacco may be enough for them; but it is not enough for me.

We must be satisfied with our condition or not satisfied. If satisfied, it is doubtful if progress will be made. We are not generally contented. The element of dissatisfaction is placed in our minds. That is our warrant of progress. And if we reform our diet, we must move out of our old habits. One by one will they be swept away, and we must move out of the lower habits into the higher.

Can a man be made savage and cruel by his diet? Can he be made stupid by his smoke? Can he be made nervously susceptible by drink? And by this combination of habits be ready to commit all petty meannesses on other individuals? If diet and drink have their effect on individuals, why not on a nation, which is but a congregation of individuals? And if it can be made savage, brutal, cruel, and unjust by its diet, why may it not be made courteous, kind, and just by another diet? If it, or we, can be made unhappy by a diet, why may we not be made happy by a change of diet? If our diet does not develop us, why may not another diet develop all our fine physical, and spiritual instincts? And in correlation to this, why if our diet and habits produce decay of our teeth, may not another diet better develop and conserve the teeth?

You may say that this view is not very encouraging, that it will be centuries before the time that men will learn to live aright, and avoid the sweltering habits we all know to be wrong, much less avoid those about which there are chances for varied arguments, and to wait until we again have an equable climate, before we can get into normal conditions, is a hopeless wait, and that the most we can do is to expect that in some remote era of the future, such a thing might be possible. Nevertheless, I say, there is no reason why we should not turn our faces and our arguments towards the right way.

For thousands of years we have been travelling towards the right government; murderous kings and nobles, and torturing priests have stood in the way. We look down the vista of centuries and our eyes can see no end to the cruelties that the privileged classes in the past have perpetrated; but were we at the other end of the vista, we should see it widen and broaden into this great and glorious republic, the culmination of the theories and work of the past students and patriots of unnumbered centuries.

For hundreds of years we have, as the representatives of the Christian idea, been prophesying the reign of an era of peace and good-will on this earth. Has it yet been accomplished? Is it now time to give up this idea as futile? *No!* I think I hear you all at once exclaim: We do not yet despair of that result.

And shall we despair that only in the dim future can reasonable men expect to find the era of perfect forms, harmonious characters and beautiful teeth? No! Our duty is to press onward towards the highest mark of our high calling, working both as present benefactors and as aids towards some more glorious era on this earth that we cannot yet see, but can dream of, hope for and desire for our children and our children's children. In doing this, let us turn towards the true diet, study well its conditions and its promises; study well the laws that produce health of the body and the mind. The glaciers or rivers of ice once swept down the torrid Amazon valley—once went sweeping over the whole eastern slope of this continent. Once, also, the deep waves of the ocean rolled in thunder tones over the spot where we now stand.

In some way or other change will take place as change has taken place. How and when I know not. Out of the fiery volcano and the deep sea has come arable land. No longer is this continent buried like the polar regions in everlasting ice, but verdure and blossoms abound. If the promise of the past has not been broken to the ear or to the heart, why may not the promise of the future be fulfilled also; and the denizens of this earth, slowly but surely, placing hand in hand and joining the great brotherhood of science, push their way up into the regions of peace, purity, justice, health and happiness?

